# Postabortion Contraceptive Utilization, Preferences, and Associated Factors among Women Receiving Abortion Care Services in Health Facilities of Ambo Town, Ethiopia

**DOI:** 10.1155/2022/2681478

**Published:** 2022-12-06

**Authors:** Bontu Aschale Abebe, Gizachew Abdissa, Gemechu Ganfure, Maru Mossisa

**Affiliations:** ^1^Department of Midwifery, Ambo University, Ambo, Ethiopia; ^2^Department of Pediatrics, Ambo University, Ambo, Ethiopia

## Abstract

**Background:**

The World Health Organization recommends the use of effective contraception for the prevention of unintended pregnancy and unsafe abortion. The main aim of postabortion contraceptive services is to prevent recurrent pregnancy and ultimately mitigate the associated maternal mortality.

**Objective:**

To assess postabortion contraceptive utilization (PACU) and postabortion contraceptive preferences (PACP) and the associated factors among women receiving abortion care services in Ambo town, Oromia Region, Western Ethiopia.

**Methods:**

A cross-sectional study was conducted at the health facilities in Ambo town from 22 July to 24 September 2021. The data was collected using a structured questionnaire. Bivariate and multivariable logistic regression was done to determine the factors associated with postabortion contraceptive utilization and preferences.

**Results:**

Out of 388 participants who were included in the final analysis, 262 (67.5%) had utilized postabortion contraceptives of which 173 (66%) received contraceptive methods of their primary preference. The multivariate logistic regression showed that cohabiting couples showed lower utilization (AOR = 0.15; 95% CI: 0.06-0.21; *p* value = 0.004) than married ones and planning to have an additional child within 1-3 years (AOR = 7.41; 95% CI: 2.18-11.41; *p* value = 0.005) or after 3-5 years (AOR = 6.67: 95% CI: 5.12-10.18; *p* value = 0.033) was identified to be significantly associated with postabortion contraceptive utilization. Having a secondary education level (AOR = 3.06; 95% CI: 1.54-6.07; *p* value = 0.001) and having experience of domestic violence (AOR = 2.19; 95% CI: 1.27-3.81; *p* value = 0.005) were significantly associated with unsatisfied postabortion contraceptive preference. *Conclusions and Recommendations*. About two-thirds of the women who were given abortion services received postabortion contraceptives whereas almost two-thirds of them received a contraceptive method of their primary preference. Marital status, duration before additional child planned, and being counseled on contraceptive determined postabortion contraceptive utilization. Having a secondary education level and having experienced domestic violence were significantly associated with unsatisfied PACP.

## 1. Introduction

Abortion is the spontaneous or induced termination of pregnancy before fetal viability [1]. The World Health Organization defines “Unsafe abortion as a procedure for terminating an unwanted pregnancy either by persons lacking the essential skills or in a setting lacking the minimal medical standards, or both” [2]. The risk of dying from an unsafe abortion was the highest in Africa [3]. Each year, between 4.7% and 13.2% of maternal deaths can be attributed to unsafe abortion [4]. In East Africa specifically, maternal mortality attributable to unsafe abortion is 18%, with the highest abortion-related maternal mortality in the world [5].

In Ethiopia, about 19.6% of maternal deaths are due to complications of abortion, and 8.6% are due to unsafe abortion [6, 7]. Millions of women suffer long-term health consequences of infertility and pelvic organ injury and thousands die subsequently after an unsafe abortion [8].

Around 7 million women are admitted to hospitals every year in developing countries, as a result of unsafe abortions [9]. An estimated 620,300 induced abortions were performed in Ethiopia, and an estimated 294,100 of which occurred outside of health facilities. The number of women receiving treatment for complications from induced abortion was about 103,600 [5].

Postabortion contraceptive is the initiation and use of contraceptive methods at the time of management of abortion or before fertility returns after the abortion within 10 to 11 days of abortions or miscarriage [10]. The WHO also recommends spacing at least 6 months between abortions, to reduce the chances of low birth weight, premature birth, and maternal anemia [11]. Contraceptive usage reduces maternal deaths by 44%, and resolving the unmet need for contraceptives could prevent 29% of maternal deaths.

The annual cost of treating major complications from unsafe abortion is estimated at 553 million US$ [12]. Low socioeconomic status women are less likely to receive high-quality counseling than their counterparts. High-quality counseling appears to reinforce preferences for effective contraception [13]. The main aim of postabortion contraceptive services is to optimize the utilization of postabortion family planning and, thus, to reduce recurrent pregnancy and ultimately reduce the associated maternal mortality. So, postabortion time is the precise time to introduce contraceptive advice because women are more prepared to accept postabortion contraceptives [10, 14].

However, there is no similar study conducted in the study area, and most of the previous studies conducted in Ethiopia on the issue involved a single health facility, particularly, hospitals, and thus, have overlooked the inclusion of health centers where a significant number of women visit first for abortion services. This study is aimed at determining the proportion of utilization, preferences of postabortion contraceptives, and the associated factors.

## 2. Methods

### 2.1. Study Setting and Period

The study was conducted in Ambo town, West Shewa Zone, Oromia, Ethiopia. Ambo town consists of 22 districts and six kebeles. Ambo is located 114 kilometers away from Addis Ababa. According to the Ambo Health Town Administration office in 2020/2021 E.C, the total population of the town is 97,317 of which 49,602 are males and 47,715 are females. In the town, there is one university, one referral hospital, one general hospital, two health centers, and 21 medium private clinics, all these facilities give postabortion contraceptive with 98% of potential health services coverage. All public health institutions and most of the private clinics are providing 24 hours of postabortion care services in town. The study was conducted from 22 July to 24 September 2021.

### 2.2. Study Design

An analytical cross-sectional study design was conducted.

### 2.3. Study Population

All women who received abortion care services during the study period were considered for the study.

#### 2.3.1. Inclusion Criteria

- Women who received abortion care services at health facilities in Ambo town during the study period who gave an informed written consent

#### 2.3.2. Exclusion Criteria

- Women who could not make the interview (critically sick, unable to hear or speak)

### 2.4. Sample Size Determination and Sampling Techniques

#### 2.4.1. Sample Size Determination

The required sample size was calculated by using single population proportion formula using the postabortion contraceptive utilization of 53.7% from a study done in Arsi in the Oromia region [15]; marginal error (*d*) of 5%, and with the constant of a standard distribution (*z*) value = 1.96 at 95% confidence level CI.

(1) $$\begin{array}{c} n=\frac{{\left(Z_{\alpha /2}\right)}^{2}p\left(1-p\right)}{d^{2}},\\ n={\left(1.96\right)}^{2}*\frac{0.54\left(1-0.54\right)}{0.05^{2}},\\ n=382. \end{array}$$

*n* is the sample size, *p* is the proportion, *d* is the marginal error, and *Z*_*α*/2_ is the critical value of the normal distribution at *α*/2 (for a confidence level of 95%, *α* is 0.05, and the critical value is 1.96).

By adding 10% for nonresponse, *n* = 10%∗382 = 38.

Thus, the final sample size was 382 + 38 = 420.

#### 2.4.2. Sampling Technique

All of the four governmental health facilities in Ambo town, a general hospital, a referral hospital, and two health centers, were purposely included, and six (30%) of the private clinics were selected using a random sampling technique. Then, systematic random sampling was used to select the study respondents in each facility.

### 2.5. Variables of the Study

#### 2.5.1. Dependent Variables

These variables were derived primarily from the study title and objectives. Then, we classified and operationalized the two variables as follows:

- Postabortion contraceptive utilization: the proportion of women who used a contraceptive method during the current abortion services in the selected health facilities
- Postabortion contraceptive preference: the contraceptive method of the primary choice by the client, with an attached personal reason or without any specific reason

#### 2.5.2. Independent Variables

We used a systematic review of the literature to devise a comprehensive list of the independent variables. Then, the variables were classified into socioeconomic, reproductive health, health service, and personal factors in line with the literature [16].

- Sociodemographic factors (age, marital status, religion, residence of mothers, ethnicity, educational status, occupation, and monthly income)
- Reproductive health factors (gravidity, parity, fertility plan, previous abortion history, contraceptive history, type of abortion, pregnancy plan, and gestational age)
- Health service-related factors (counselling, distance to facility, type of health facility, time of counseling, type of procedure, and room of contraceptive received)
- Personal factors (disagreement with husband, knowledge of fertility, knowledge of contraceptives, number of living children, and electronic media)

### 2.6. Data Collection Tools and Techniques

A structured questionnaire was adapted and modified from the related previous study [16], and then, questions intended to determine the preferences of postabortion contraceptive methods as well as factors not included in the previous study were added. The questionnaire consisted of six sections including clients' and facilities' characteristics, knowledge of contraceptives, postabortion contraceptive utilization, and preferences. The questionnaire was prepared in English translated into the Afan Oromo language and translated back to English to keep the consistency of the question. The data was collected through interviews using a structured questionnaire after the healthcare provider decided to discharge the women before they leave health facilities. Data was collected through a face-to-face interview involving 10 data collectors (nurses or midwives).

### 2.7. Data Quality Control and Management

To ensure data quality, a 2-day training was given to the data collectors by the principal investigator to make them familiar with the data collection tools, the study objectives and significance, interviewing techniques, the purpose of the study, and the importance of privacy and approach to the interviewees. The data collection tool was pretested on 22 (5%) women on abortion services from 19 to 21 July 2021 in the selected 10 health facilities. However, the data was later excluded from the main study. And necessary changes were made to the questionnaire before starting the main study. The principal investigator was making day-to-day supervision of the data collection process checking the completeness of the filled questionnaires every day and timely responding to any difficulties faced by the data collectors.

### 2.8. Data Processing and Analyzing

Data were entered and cleaned using the latest available Epi info version 7.2.3.1. The cleaned data were exported and analyzed by SPSS version 25.0. Descriptive statistics, using frequency, and percentages with tables and charts were used to analyze the proportion of utilization and preferences of postabortion contraceptive use and the specific factors whereas bivariate and multivariate logistic regression was used to determine the associated factors. All variables with *p* value < 0.25 during bivariate logistic regression analysis were considered for multivariate logistic regression. All analysis was done with a 95% confidence interval, and significance was considered with *p* value less than 0.05.

### 2.9. Ethical Consideration

Ethical clearance was obtained from the institutional review board of the College of Medicine and Health Sciences of Ambo University. The site clearance was obtained from the administrators of each of the ten health facilities. Finally, written informed consent was obtained from each of the participants after explaining the purpose and procedure of the study. All information is collected and documented anonymously, and the participant's confidentiality was protected throughout the study period.

## 3. Results

### 3.1. Sociodemographic Characteristics

A total of 420 women who received abortion services at health facilities in Ambo town were approached to be included in this study. However, 32 patients were excluded from the final analysis including 23 patients who declined to give informed consent and 8 who withdrew before completing the interview. Thus, a total of 388 women were included in this study; achieving a response rate of 92.2%. A total of 222 (57.2%) participants were youths 15 to 24 years of age. Nearly half 188 (48.4%) of the respondents were married; 317 (81.7%) were from the Oromo ethnic group, 304 (78.4%) were urban dwellers, and 46 (11.9%) had no formal education. One hundred and twenty-two (31.5%) were students (Table 1).

### 3.2. Institutional, Personal, and Reproductive Characteristics of the Participants

A total of 311 (80.2%) participants had no history of abortion whereas only 124 (32%) of the current pregnancies were planned. A total of 253 (65.2%) of the women received SAC services whereas rape and incest accounted for 161 (63.6%) of the reasons for SAC. A total of 278 (71.6%) of the abortion services employed medication use. Over half (56.4%) of the participants had previously used contraceptives, and 123 (31.7%) had ever experienced domestic violence from a partner. Another 276 (71.4%) participants were counseled on contraceptive use during the current visit, and 229 (59.1%) had more than average knowledge of contraceptives. While 206 (53.1%) received service on payment, 95 (24.48%) women had ever been discouraged from contraceptive use by a partner (Table 2).

### 3.3. Postabortion Contraceptive Utilization and Preferences

**Figure 1:**
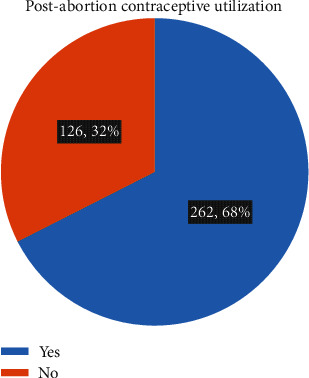
Postabortion contraceptive utilization among women who received abortion care services at health facilities in Ambo town, Oromia region, Ethiopia, 2021.

Out of 388 participants, 262 (67.5%) received postabortion contraceptives (Figure 1).

**Figure 2:**
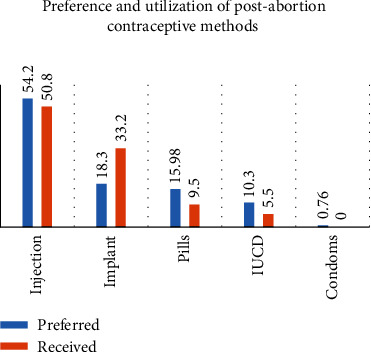
The primary preferences and utilization of postabortion contraceptive methods among women who received abortion care services at the health facilities in Ambo town, Oromia region, Ethiopia, 2021.

Out of 262 women who received postabortion contraceptives, 173 (66%) received contraceptive methods of their primary preference. Injections were primarily preferred by 142 (54.2%) participants followed by implants that were primarily preferred by 48 (18.3%) of them. Only 41 (15.6%) and 27 (10.3) participants preferred pills and intrauterine contraceptive devices (IUCD), respectively. Among the reasons for their preferences for postabortion contraceptive methods, being short-acting and the safety of the method were the most frequently reported, by 77 (29.39%) participants each. The longer duration of action of the method was reported as the main reason that influenced their preferences by 66 (25.19%) participants (Figure 2).

### 3.4. Factors Associated with Postabortion Contraceptive Utilization

#### 3.4.1. Bivariate Logistic Regression

The bivariate logistic regression showed marital status, the duration before having an additional child, gestational age of current pregnancy, being counseled about a contraceptive, experience of any domestic violence, being discouraged on contraceptive use by partner, any health problem, and knowledge on contraceptive were significantly associated with PACU.

#### 3.4.2. Multivariate Logistic Regression

The results of multivariate logistic regression analysis showed that women who were cohabiting were 85% less likely to utilize postabortion contraceptives compared to married women (AOR = 0.15; 95% CI: 0.06-0.21; *p* value = 0.004). Women who were planning to have an additional child after 1-3 years were about 7.41 times more likely (AOR = 7.41; 95% CI: 2.18-11.41; *p* value = 0.005), and women intending to have an additional child after 3-5 years were about 6.67 times more likely (AOR = 6.67; 95% CI: 5.12-10.18; *p* value = 0.033) to use postabortion contraceptive as compared to women who planned to have an additional child within one year. Women who were counseled about contraceptives during the current visit were 10.42 times more likely to accept postabortion contraceptives (AOR 10.42: 95% CI: 3.20-17.20) than women who were not counseled. Women who had any health problem were 3.40 times more likely to accept postabortion contraceptives (AOR 3.40: 95% CI: 2.14-10.21) than those who did not have any health problems (Table 3).

### 3.5. Factors Associated with Postabortion Contraceptive Preference

#### 3.5.1. Bivariate Logistic Regression

The bivariate logistic regression showed that marital status, education, occupation, monthly income, type of health facility, getting service on payment, experienced domestic violence by a partner, and any health problem were all significantly associated with PACP.

#### 3.5.2. Multivariate Logistic Regression

The results of multivariate logistic regression analysis showed that women with secondary education level were about 3.06 times more likely (AOR = 3.06; 95% CI: 1.54-6.07; *p* value = 0.001) to receive postabortion contraceptives which were not of their primary preferences compared to those with education level of college or above. Women who had experienced any domestic violence were about 2.19 times more likely (AOR = 2.19; 95% CI: 1.27-3.81; *p* value = 0.005) to receive a PAC method that was not of their primary preference compared to those who had not incurred any domestic violence (Table 4).

## 4. Discussion

In the current study, out of 388 women who were given abortion care services at health facilities in Ambo town, 67.5% (62.4%-72.2% at 95% C.I) received postabortion contraceptives. The current utilization level is comparable with the proportion of postabortion contraceptive use from previous studies in Ethiopia: 70.9% in Northern Tigray [17] and 64.8% in Felege Hiwot referral hospital [18].

The current proportion of PACU, however, was higher than the one reported by other previous studies conducted in Ethiopia: 45.8% in Debre Berhan [19], 57% in Addis Ababa [20], and 53.7% in Arsi [15]. These differences might be due to cultural, educational, and income differences that can directly or indirectly influence the perception, attitude, and practice of family planning. Another explanation might be the trend towards improvement in the quality and coverage of health services in general and family planning services in particular during the past years.

On the other hand, the current PACU proportion is lower than proportions reported by previous studies from Burayu, Oromia (88.5%) [16], South Wollo, Amhara (84%) [21], and Bahir Dar, Amhara (78.5%) [22]. This difference might be attributed to variations in cultural norms, literacy levels, and religious values of the participants. Similarly, the current utilization proportion is lower than those reported in Kenya (78.2%) [23], Tanzania (90%) [24], and Northern Brazil (97.4%) [25]. The potential reasons for these discrepancies might include the variations in cultural values, literacy, and lifestyles as well as the differences in the healthcare systems, health coverage, and contraceptive services between countries. For example, having electronic media knowledge on return of fertility and having prior information about family planning have been shown to be important determinants of PACU [18, 21, 22].

Overall, 89 (34%) of the women did not receive the postabortion contraceptive method of their primary preference. This shows that a significant number of the women did not get their first preference probably because of limited access or lack of some of the specific methods of contraception in the facilities. The inability to receive the contraceptive method of their primary preference is an indicator of little empowerment of the women in health care services [26].

In the current study, marital status is one of the factors independently associated with the utilization of postabortion contraceptives. Women with the marital status of cohabiting were 85% less likely to use postabortion contraceptives than married women. This can be explained by the fact that almost two-thirds (60.1%) of the married women used postabortion care services compared to only 23.7% of those cohabiting and possibly more of those that used postabortion care services might be more likely to receive postabortion contraceptives compared to those that used SAC services probably because most of those that presented with rape, underage, and incest as reasons of SAC did not receive postabortion contraceptive and mainly reported not having a sexual partner as a reason for not accepting it. Moreover, married women might have had more regular sex compared to those cohabiting and those cohabiting might fear the stigma to use contraceptive methods compared to those married. Two previous studies [20, 27] and a systematic review of studies from East Africa [28] also concluded that married women showed a significantly higher proportion of postabortion contraceptive utilization.

Women who were planning to have an additional child after 1-3 years were about 7.41 times more likely and those that planned to have one within 3-5 years were about 6.67 times more likely to utilize postabortion contraceptives compared to those who planned to have a child within one year. This is probably explained by the higher PACU by those who did not want to have a child so soon because of personal or medical reasons. A study conducted in Northern Tigray also showed that the time when women planned to have a child was significantly associated with PACU [29].

Women who were counseled about contraceptives during the current visit were 10.20 times more likely to accept postabortion contraceptives. This finding is consistent with previous studies [15, 18, 19, 28] that reported counseling to be an independent determinant of PACU. Counseling might avoid negative attitudes and perceptions on the effectiveness and safety of contraceptives and might optimize their knowledge and, thus, increase the PACU. Previous studies also showed that previous use of contraceptives and having knowledge of contraceptive methods were significantly associated with higher PACU [18, 20].

Women who had any health problems were 3.40 times more likely to use postabortion contraceptives than those who did not have any health problems. This is likely related to the recommendations by doctors to avoid pregnancy in women with unfavorable conditions including heart failure [30] and renal failure [31].

On the other hand, women with a secondary education level compared to college and above, and those who had experienced domestic violence were identified as factors independently associated with PACP. Women with a secondary education level were about 3.06 times more likely not to receive their first choices compared to those with an education level of college and above. This can be explained by the fact that 64.3% of the former had a monthly income level of ≤2000 ETB compared to 47.2% of those with an education level of college and above. Previous studies showed that the cost related to receiving the service, including the direct and indirect medical and nonmedical costs, is an important predictor of receiving the preferred contraceptive method [26, 32, 33].

Similarly, women who had ever experienced any domestic violence by their partner were about 2.19 times more likely to receive a postabortion contraceptive that was not of their primary preference compared to those who had not incurred any domestic violence. These women might be influenced by the choice of their partners or may not get adequate support and understanding from them to receive their preferred postabortion contraceptive methods. Using a preferred method is an indicator of access to care and reproductive autonomy [26].

## 5. Limitation and Strengths

### 5.1. Strengths

- Both public and private health facilities have different levels of health care including clinics, health centers, general hospitals, and referral hospitals
- Postabortion contraceptive preference was also studied in addition to the utilization

### 5.2. Limitations

- Some questions were very sensitive for some of the women, and a few of them responded to them with difficulty; others withdrew from the study despite the use of techniques to minimize such social desirability biases

## 6. Conclusions

About two-thirds of the women who received abortion services received postabortion contraceptives. Overall, the primary preference of about one-third of the women was not met. Marital status, plan to have an additional child, being counseled on contraceptives, and having a comorbid health problem were shown to be significantly associated with postabortion contraceptive utilization. Finally, education level and having experienced domestic violence were identified as factors independently associated with postabortion contraceptive preference.

## Figures and Tables

**Table 1:** The sociodemographic characteristics of women who received abortion care services at health facilities in Ambo town, Oromia region, Ethiopia, 2021.

Variable	Category	Frequency	Percentage
Age	15-24	222	57.2
	25-34	135	34.8
	≥35	31	8.0
Marital status	Married	188	48.4
	Single	135	34.8
	Separated	27	7.0
	Cohabiting	38	9.8
Ethnicity	Oromo	317	81.7
	Amhara	66	17.0
	Others^a^	5	1.3
Religion	Protestant	188	48.5
	Orthodox	165	42.5
	Others^b^	35	9.0
Place of residence	Urban	304	78.4
	Rural	84	21.6
Level of education	No formal education	46	11.9
	Primary	100	25.8
	Secondary	115	29.6
	Collage or above	127	32.7
Occupation	Self-employed	85	21.9
	Unemployed	94	24.2
	Employed	87	22.4
	Student	122	31.5
Income level^c^ (ETB)	≤2000	216	55.7
	2001-4000	84	21.6
	>4000	88	22.7

^a^Tigre, Somali, Gurage. ^b^Muslim, Waqefata, and Catholic. ^c^Classification according to the forecast of per capita income of Ethiopia by Trading Economics, 2021.

**Table 2:** Institutional, personal, and family factories of women who received abortion care services at health facilities in Ambo town, Oromia region, Ethiopia, 2021.

Variable	Category	Frequency	Percentage
History of abortion	Yes	77	19.8
	No	311	80.2
Condition of current pregnancy	Planned	124	32
	Unplanned	264	68
Type of service received	SAC	253	65.2
	PAC	132	34.8
The reason for SAC (*n* = 253)	Rape and incest	161	63.6
	Maternal indication	63	24.9
	Others^a^	29	11.5
Method of abortion care applied	Medication	278	71.6
	Manual vacuum aspiration	88	22.68
	Mixed	18	4.6
	Spontaneous	4	2.06
The reason for PAC (*n* = 135)	Taking the drug without prescription	38	28.2
	Prescribed by a traditional healer	11	8.1
	Spontaneous	78	57.8
	RH negative	8	5.9
Previous use of contraceptive history	Yes	219	56.44
	No	169	43.6
Experienced any domestic violence from partner/husband	Yes	123	31.7
	No	265	68.29
Sex of health provider	Male	224	57.7
	Female	164	42.3
Any health problem	Yes	111	28.6
	No	277	71.39
Counseled about contraceptive	Yes	276	71.1
	No	112	28.86
Type of health facility	Public	197	50.77
	Private	191	49.22
Knowledge on contraceptives	Yes	229	59.02
	No	159	40.9
Partner ever discouraged contraceptive use	Yes	95	24.48
	No	293	75.51
Service received by payment	Yes	206	53.06
	No	182	46.93

^a^Fetal deformity or underage.

**Table 3:** Bivariate and multivariate logistic regression for the factors associated with postabortion contraceptive utilization among women who received abortion care services at health facilities in Ambo town, Oromia region, Ethiopia, 2021.

Variables	Categories	Postabortion contraceptive use		COR (95% C.I)	AOR (95% C.I)	*p* value
		Yes	No			
		Frequency (%)	Frequency (%)			
Marital status	Married	135 (71.8)	53 (28.2)	1	1	
	Single	90 (66.7)	45 (33.3)	0.79 (0.49-1.27)	0.86 (0.19-2.64)	.252
	Separated	16 (59.3)	11 (40.7)	0.57 (0.25-1.31)	4.73 (0.69-19.66)	.121
	Cohabiting	21 (55.3)	17 (44.7)	0.49 (0.24-0.99)	0.15 (0.06-0.21)	.004
Time planned to have an additional child	In one year	22 (46.8)	25 (53.2)	1	1	
	1-3 years	55 (82.1)	12 (17.9)	5.21 (2.23-12.16)	7.41 (2.18-11.41)	.005
	3-5 years	49 (68.1)	23 (31.9)	2.42 (1.14-5.16)	6.67 (5.12-10.18)	.043
	After five years	26 (57.8)	19 (42.2)	1.56 (0.68-3.54)	4.23 (0.65-8.27)	.229
Gestational age of current pregnancy	<9 weeks	163 (74.1)	57 (25.9)	3.32 (1.80-6.13)	5.04 (0.83-30.80)	.095
	9-12 weeks	74 (64.9)	40 (35.1)	2.15 (1.11-4.15)	1.67 (0.19-15.05)	.517
	>12 weeks	25 (46.3)	29 (53.7)	1	1	
Counseled about contraceptive	Yes	211 (77.6)	61 (22.4)	4.41 (2.77-7.01)	10.20 (3.94-17.20)	<.001
	No	51 (44.0)	65 (56.0)	1	1	
Experienced any domestic violence	Yes	89 (72.4)	34 (27.6)	1.39 (0.87-2.23)	0.91 (0.18-2.26)	.714
	No	173 (65.3)	92 (34.7)	1	1	
Partner ever discouraged contraceptive use	Yes	56 (58.9)	39 (41.1)	1	1	
	No	206 (70.3)	87 (29.6)	1.65 (1.02-2.66)	2.72 (0.75-6.39)	.149
Any health problem	Yes	81 (73.0)	30 (27.0)	1.43 (0.88-2.33)	3.40 (2.14-10.21)	.024
	No	181 (65.3)	96 (34.7)	1	1	
Knowledge on contraceptive	Yes	164 (71.6)	65 (28.4)	1.57 (1.02-2.41)	0.49 (0.29-2.80)	0.251
	No	98 (61.6)	61 (38.4)	1	1	

**Table 4:** Bivariate and multivariate logistic regression for the factors associated with postabortion contraceptive preference among women who received abortion care services at health facilities in Ambo town, Oromia region, Ethiopia, 2021.

Variables	Categories	Preference		COR (95% C.I)	AOR (95% C.I)	*p* value
		No	Yes			
		Frequency (%) *N* = 89	Frequency (%) *N* = 173			
Marital status	Married	39 (28.9)	96 (71.1)	1	1	
	Single	36 (40.9)	52 (59.1)	1.73 (0.97-3.00)	1.55 (0.76-3.16)	.233
	Separated	4 (22.2)	14 (77.8)	0.70 (0.22-2.27)	.91 (0.5-3.34)	.884
	Cohabiting	10 (47.6)	11(52.4)	2.24 (0.88-5.69)	1.73 (0.59-5.09)	.321
Educational level	No formal education	9 (26.5)	25 (73.5)	1.05 (0.43-2.58)	1.12 (0.45-2.81)	.806
	Primary	21 (29.2)	51 (70.8)	1.20 (0.59-2.42)	1.20 (0.59-2.44)	.618
	Secondary	37 (52.9)	33 (47.1)	3.26 (1.66-6.40)	3.06 (1.54-6.07)	.001
	College or above	22 (25.6)	64 (74.4)	1	1	
Occupation	Self-employed	17 (30.9)	38 (69.1)	1	1	
	Unemployed	22 (33.8)	43 (66.2)	1.14 (0.53-2.47)	1.42 (0.62-3.25)	.410
	Employed	14 (25.5)	41 (74.5)	.76 (0.33-1.76)	.69 (0.27-1.78)	.445
	Student	36 (41.4)	51 (58.6)	1.58 (0.77-3.22)	1.50 (0.68-3.30)	.317
Income level	≤2000	18 (33.3)	36 (66.7)	2.33 (1.16-4.68)	2.12 (0.99-4.54)	.054
	2001-4000	13 (21.7)	47 (78.3)	1.81 (0.78-4.17)	1.35 (0.56-3.27)	.508
	≥4001	89 (34.0)	173 (66.0)	1	1	
Type of health facility	Public	37 (28.5)	93 (71.5)	1	1	
	Private	52 (39.4)	80 (60.6)	1.63 (0.97-2.74)	1.01 (0.24-4.31)	0.985
Payment to get service	Yes	55 (40.4)	81 (59.6)	1.84 (1.09-3.10)	1.32 (0.75-2.31)	.341
	No	34 (27.0)	92 (73.0)	1	1	
Experienced any domestic violence	Yes	42 (47.2)	47 (52.8)	2.40 (1.40-4.09)	2.19 (1.27-3.81)	0.005
	No	47 (27.2)	126 (72.8)	1	1	
Any health problem	Yes	34 (42.0)	47 (58.0)	1.66 (0.96-2.85)	1.32 (0.72-2.43)	0.373
	No	55 (30.4)	126 (69.6)	1	1	

## Data Availability

The datasets used and analyzed during the current study are available from the corresponding author on reasonable request.

## Abbreviations

AOR: Adjusted odds ratio
CI: Confidence interval
COR: Crude odds ratio
IUCD: Intrauterine contraceptive device
PACP: Postabortion contraceptive preference
PACU: Postabortion contraceptive utilization
SAC: Safe abortion care
SPSS: Statistical Package for Social Science
US: United States
WHO: World Health Organization.
